# Genomes of nine cultured microbes from two hydrologically connected freshwater sites in Wellesley, MA

**DOI:** 10.1128/mra.00861-24

**Published:** 2024-12-20

**Authors:** Sasha Behar, Arina Zadvornaya, Elizabeth Wren, Marina Andreadis, Ayesha Tariq, Mary E. Tian, Sachi Krishna, Kadidia Keita, Helena Eich, Jennifer Enriquez, Britany J. Hansten Monterrozo, Charlotte Hyun, Kara L. Jenkins, Kavya Parameswar, Malika M. Top, Stella Waag, M. Gray Ryan, Ashley M. Stein, Steven J. Biller

**Affiliations:** 1 Department of Biological Sciences, Wellesley College, Wellesley, Massachusetts, USA; Loyola University Chicago, Chicago, Illinois, USA

**Keywords:** freshwater, genome assembly

## Abstract

We present the genomes of nine cultured microbes isolated from two freshwater sites in Wellesley, MA. The dataset is useful for exploring genomic diversity among freshwater taxa, including *Pedobacter*, *Pseudomonas*, *Rhodoferax*, *Rouxiella,* and *Flavobacterium*.

## ANNOUNCEMENT

Bacteria play essential roles in driving the biogeochemical cycles of freshwater ecosystems (1). To explore patterns of genomic diversity and intraspecific variation within freshwater microbial communities, we sequenced a set of heterotrophic bacteria isolated from two lakes on the suburban campus of Wellesley College (Wellesley, MA, USA). Bacteria were cultured from two hydrologically connected sites: Paintshop Pond (42.291970 °N, 71.315296 °W) and downstream within the larger Lake Waban (42.289708 °N, 71.302789 °W). Paintshop Pond was the site of the former Henry Wood’s Sons paint factory, which manufactured paint pigments from 1848 until 1910. This activity left a history of significant metal and cyanide contamination at the site that was remediated between 2000 and 2003. Surface water samples from the two sites were spread onto either R2A (2) or PS (3) agar plates and grown for 2 days at 15°C. Individual colonies were purified by re-streaking on a fresh plate, then grown in the same liquid medium.

DNA was extracted using the NEB Monarch Genomic DNA Purification Kit with no size selection. Long-read sequencing libraries were constructed using the Oxford Nanopore Rapid Barcoding Kit V14 (RBK114.24) and sequenced on an Oxford Nanopore MinION R10.4.1 flow cell, yielding 12,694–169,049 reads per library with mean lengths ranging from 3,702 to 12,200 bp (Table 1). Demultiplexing, adapter trimming, and basecalling was carried out using the SUP model in Dorado (v0.7.0) (https://github.com/nanoporetech/dorado). Additional Illumina sequencing was conducted for a subset of samples with low Nanopore sequence yield. Illumina libraries were prepared with the Illumina DNA Prep tagmentation kit and IDT For Illumina Unique Dual Indexes, and then sequenced on an Illumina NextSeq2000 (2 × 150 bp reads, 2.9–3.5 million reads per library) by SeqCoast Genomics.

**TABLE 1:** Genome assembly summary data

Strain	Sampling location	Isolation media	Sequencing methodology	Nanopore reads	Nanopore read N50 (bp)	Nanopore coverage (x)	Illumina read pairs	Illumina coverage (x)	Total contigs	Plasmid contigs	Circular/ complete?	Assembly length (bp)	Assembly N50 (bp)	GC content (%)	CheckM2 completeness (%)	CheckM2 contamination (%)	Total genes	Total CDS	GTDB taxonomy (genus)	Assembly accession	SRA raw data accession (long read, short read)
WC2401	Paintshop Pond	PS	Nanopore	24,963	8,026	20			2	1		5,202,312	5,197,844	59	100	0.94	4779	4677	Pseudomonas_E	GCA_041200115.1	SRR29831499
WC2409	Paintshop Pond	R2A	Nanopore + Illumina	12,694	14,773	19	3,320,938	254	1	0		3,915,292	3,915,292	33	99.99	0	3352	3274	Flavobacterium	GCA_041200085.1	SRR29831498, SRR29831487
WC2416	Paintshop Pond	R2A	Nanopore	24,726	19,978	52			1	0		3,912,994	3,912,994	33	99.94	0	3325	3247	Flavobacterium	GCA_041200095.1	SRR29831494
WC2420	Lake Waban	PS	Nanopore	169,049	8,443	118			2	1	Yes	5,283,909	5,274,856	50	100	0.48	4870	4755	Rouxiella	GCA_041200025.1	SRR29831493
WC2421	Lake Waban	PS	Nanopore + Illumina	15,337	19,142	32	2,854,856	223	1	0	Yes	3,833,975	3,833,975	33	99.92	0	3269	3191	Flavobacterium	GCA_040822115.1	SRR29831492, SRR29831497
WC2423	Lake Waban	PS	Nanopore	94,446	8,700	75			1	0	Yes	5,978,383	5,978,383	39	99.99	0.39	5037	4951	Pedobacter	GCA_040822065.1	SRR29831491
WC2427	Paintshop Pond	PS	Nanopore	45,460	9,488	51			1	0	Yes	4,778,649	4,778,649	64	100	1.06	4434	4376	Rhodoferax_B	GCA_040822085.1	SRR29831490
WC2429	Paintshop Pond	PS	Nanopore + Illumina	18,563	24,113	53	3,457,624	257	1	0		4,043,312	4,043,312	33	99.75	0.11	3466	3388	Flavobacterium	GCA_041200105.1	SRR29831489, SRR29831496
WC2430	Paintshop Pond	PS	Nanopore + Illumina	16,324	26,742	51	2,869,147	221	1	0	Yes	3,896,973	3,896,973	33	99.99	0.03	3392	3314	Flavobacterium	GCA_040822075.1	SRR29831488, SRR29831495

All genome assembly steps were performed using the KBase platform (4). Nanopore sequences were filtered using Filtlong (v0.2.1) (https://github.com/rrwick/Filtlong), and Illumina sequences were trimmed using Trimmomatic (v0.36) (5), both with default parameters. Genomes were assembled using Flye (v2.9.4) (6) and, when available, polished with Illumina data using Polypolish (v0.6.0) (7) (Table 1). Taxonomy was assigned to the genus level using GTDB-Tk (v1.7.0) (8) against the GTDB r202 database release. Assembled contigs were checked for circularity, cleaned, and rotated where applicable with Circlator (v1.5.5) (9). Genome completeness was estimated using CheckM2 (v1.0.2) (10). Putative plasmids were identified using geNomad (v1.8.0) (11), and genomes were annotated by NCBI using the Prokaryotic Genome Annotation Pipeline (v6.7) (12).

We obtained nine high quality genome assemblies, ranging between 3.83 and 5.98 Mbp in length and 33%–64% GC (Table 1). All sequences are estimated to be >99% complete and contain <1.1% contamination. Five genomes were complete and closed; the other four assembled into a single chromosomal contig each but could not be confirmed as circular. Five of these genomes represent members of the genus *Flavobacterium*, including colonies isolated from both sampling locations and on the two different isolation media. The other four strains belong to the genera *Pedobacter*, *Pseudomonas*, *Rhodoferax*, and *Rouxiella* (Table 1).

## Data Availability

All sequence data are available under NCBI BioProject PRJNA1134181 and *via* the specific accession numbers listed in Table 1. Data analyses can be accessed through KBase *via* the overview narrative at https://doi.org/10.25982/186528.9/2404396.
